# Polish Cultural Adaptation and Reliability of the Fugl-Meyer Assessment of Motor Performance and Sensory Assessment Scale in Stroke Patients

**DOI:** 10.3390/jcm13133710

**Published:** 2024-06-26

**Authors:** Magdalena Goliwąs, Joanna Małecka, Katarzyna Adamczewska, Marta Flis-Masłowska, Jacek Lewandowski, Piotr Kocur

**Affiliations:** Department of Clinical Physiotherapy, University School of Physical Education, 61-871 Poznan, Poland; malecka@awf.poznan.pl (J.M.); adamczewska@awf.poznan.pl (K.A.); flis@awf.poznan.pl (M.F.-M.); lewandowski@awf.poznan.pl (J.L.); pkocur@awf.poznan.pl (P.K.)

**Keywords:** stroke, fugl-meyer assessment, reliability, translation, upper and lower limb, sensory

## Abstract

**Background and Purpose**: The Fugl-Meyer Assessment of Motor Performance and Sensory Assessment Scale (FMA) is the most commonly used and recommended outcome measure for the sensorimotor impairment of the upper and lower limbs in stroke patients. The aim of this study was to perform cross-cultural translation and adaptation of the scale into Polish and to evaluate the FMA’s reliability of motor performance and sensation of the upper and lower limb sections among ischemic stroke patients. **Methods**: The Polish version of the FMA (FMA-PL) was developed using a forward–backward translation performed by a group of experts and then evaluated by a panel of judges according to international guidelines. The study involved 86 patients (F = 30, M = 56, i.e., 35%; the average age of patients was 64 ± 12 years, 36 with right-sided stroke and 50 with left-sided stroke). The FMA-PL was carried out twice by two experienced neurological physiotherapists with a 2 h gap between assessments (test–retest and inter-rater). The reliability of the outcome measure was defined by calculating the intraclass correlation coefficient (ICC). The standard error of measurement (SEM) and the minimum detectable change (MDC) were also calculated. The internal consistency of the test was determined by the Cronbach’s alpha indicator. **Results**: Three domains were evaluated on the FMA-PL scale. From the whole test, results were obtained in the range of 12–124 points: 64 points for FMA-UE-PL 2, 34 points for FMA-LE-PL 4, and 24 points for FMA-S-PL 0. The ICC values were in the range of 0.99–1.00 for the total FMA-PL score and the results of each domain. The SEM and MDC for the entire FMA-PL calculated for test–retest measurements were 0.22 and 1.60, respectively. The SEM and MDC for the total FMA-PL score obtained during repeated measurements of the same investigator were 1.3 and 3.5 points, respectively. The Cronbach’s alpha values calculated for the total FMA-PL, FMA-UE-PL, FMA-LE-PL, and FMA-S-PL items amounted to 0.938–0.939, 0.932–0.934, and 0.634–0.722, respectively. **Conclusions**: The Polish version of the FMA is a consistent and reliable outcome measure for the motor and sensory evaluation of the upper and lower limbs for patients in subacute and chronic stroke stages.

## 1. Introduction

In Poland, stroke is the third leading cause of death, after other cardiovascular diseases and cancer [1,2,3,4,5,6]. In the majority of stroke survivors, difficulty in maintaining balance in sitting or standing positions and limitations of the motor efficiency of the limbs are observed. Moreover, an uneven distribution of the plantar pressure forces of the side of the feet on the ground is present. In the late post-stroke period, 25 to 74% of patients have problems with activities of daily living (ADL), such as eating, grooming, and mobility [7,8,9,10,11,12].

During the physiotherapeutic process, one of the primary objectives of the stroke patient’s physical assessment is to evaluate motor and sensory functions. For this reason, the use of outcome measures is necessary to enable the assessment of patient performance and therapeutic progress in a useful, objective, and reliable way. Therefore, there is a great need for the cultural adaptation of the gold standard outcome measures and their implementation during research and clinical work in Poland. The outcome measures can be used for the sensorimotor assessment of the Polish stroke population by neurologists, physiotherapists, and occupational therapists. This will allow the determination of the patient’s functional improvement and inform the patient, family, or institution paying for the treatment process about the results. The Fugl-Meyer Assessment (FMA) is a clinical outcome measure recommended for stroke patients, inter alia, by the American Physical Therapy Association—Neurology Section. The FMA obtained the highest recommendation score of 4 for the evaluation of the lower limbs. This means that the test has excellent psychometric properties for the stroke population. The upper limb evaluation has been recommended with as high a score of 3 and this means that the test has good clinical utility. The sensory evaluation section is not recommended for clinical trials but has equally important educational values [13,14].

This scale was first proposed by Axel Fugl-Meyer in 1975. The questionnaire has been translated, validated, and culturally adapted into many languages: Spanish [15,16], Danish [17], Italian [18], and Romanian [19]. Translation and cultural adaptation of the test are more frequently performed for domains/parts assessing the motor skills of the upper and lower limbs. Few scientists have validated the sensory assessment part [14,15,16,17,18,19]. The motor part of the FMA has been used extensively [20,21,22,23] and has shown excellent reliability, validity, and responsiveness [24,25,26]. The motor domains of the FMA are recommended to evaluate the level of sensorimotor function and the progress of motor recovery [22,27].

The aim of this study was a cross-cultural translation and adaptation of the FMA-PL motor and sensory part for the upper and lower limbs and to determine its reliability among an ischemic stroke patient population.

## 2. Methods

### 2.1. Study Design, Participants, Initial Evaluation

This was a cross-sectional study. The research was carried out parallelly in the department of stroke treatment in Poznań and in the department of acute neurological rehabilitation in Piaski. The inclusion criteria comprised diagnosed stroke (diagnosis based on computed tomography or magnetic resonance imaging), hemiplegia, the absence of additional orthopedic or neurological disorders causing disability, and an age of over 18 years. Patients of both sexes were examined. Exclusion criteria were inability to understand the instructions, the presence of serious vision problems, a mother tongue other than Polish, severe cognitive impairments, cerebellar damage, peripheral neuropathy, and hemorrhagic stroke. For the initial assessment, the collected information included demographic data such as age, gender, weight, height, and lateralization. Clinical data such as the duration of illness, the involved side of stroke, the presence of concomitant diseases, and the duration of the rehabilitation program in the hospital were also collected. The study was approved by the Bioethical Commission of Poznań University of Medical Science (Resolution Number 413/17) and also was conducted in accordance with the Declaration of Helsinki. We obtained permission to translate the FMA scale into Polish from the research group of Rehabilitation Medicine at the University of Gothenburg, which is the curator of the original FMA scale developed by Axel Fugl-Meyer in 1975. Informed consent was obtained from all participants at the time of registration for the study. The study ultimately involved 86 people. One person did not participate in the retesting and therefore was rejected. The study lasted 12 months. The sensorimotor evaluation of patients was carried out by two researchers, physiotherapists, who had been working with patients with neurological deficits at various stages of improvement for at least 5 years.

### 2.2. Outcome Measures

The original outcome measure proposed by Fugl-Meyer [14] assesses 5 components/domains, including motor function for the lower extremities (FMA-LE, maximum score 34) and for the upper extremities (FMA-UE, maximum score 66), sensory function (FMA-S, maximum score 24), balance (maximum score 14), joint range of motion (maximum score 44), and joint pain (maximum score 44). In this study the evaluation of 3 domains was made: FMA-LE, FMA-UE, and FMA-S. The FMA-LE domain assesses 6 categories of movements: basic reflex activity in the lower limb, volitional movement with muscle synergies in different positions, coordination, and speed of movement (tremor, dysmetria, time). For the majority of tasks, points are awarded from 0 to 2, where 0 means the movement is not performed, 1 means a partial movement, and 2 means the movement is performed infallibly. In the FMA-UE domain, 9 categories and movements are evaluated: primary reflexes in the upper limb, flexor synergy, extensor synergy, synergy-combining movements, non-synergy movements, wrist movements, hand movements, and coordination assessment (tremor, dysmetria, speed). For most tasks, a score of 0 to 2 is awarded, where 0 means the movement is not performed, 1 means a partial movement, and 2 means the movement is performed infallibly. In the FMA-S domain, the feeling of touch is assessed in the area of the shoulder, arm, thigh, and sole of the foot. Points are awarded as follows: 0—lack of sensation; 1—hyperesthesia; 2—correct feeling. In addition to the feeling of touch, proprioceptive senses are also tested during movement of the shoulder, elbow, wrist, thumb, hip, knee, ankle, and toe, and the score is also given from 0 to 2. The maximum score for the total motor and sensory scale is 124 points (66 for FM-UE, 34 for FM-LE, and 24 for FMA-S) [28,29,30].

### 2.3. Stages of Translation and Adaptation

**Figure 1:**
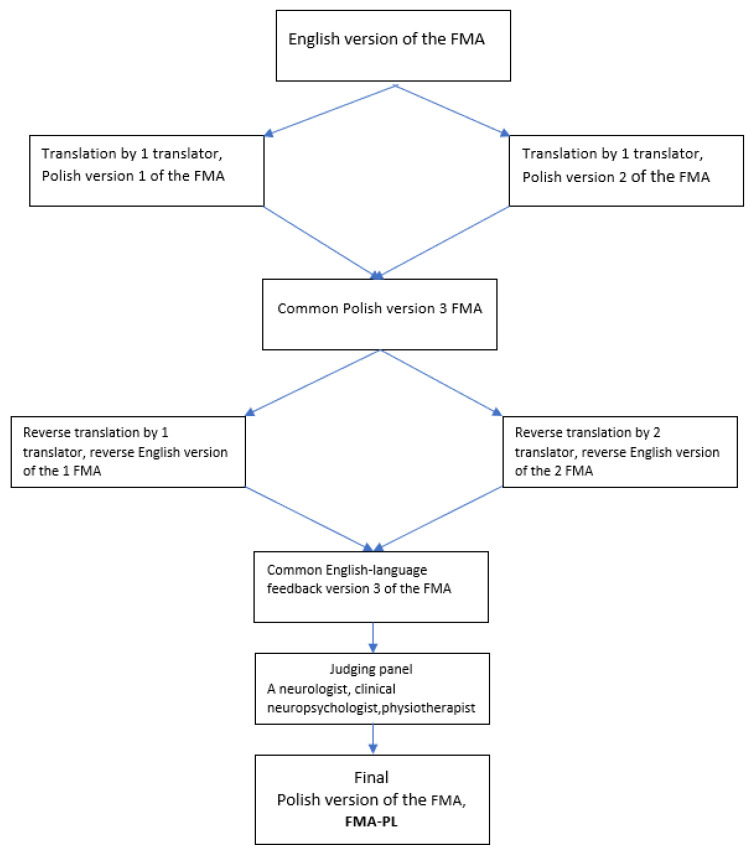
Graphical representation of the translation process.

The first stage was the translation of the Fugl-Meyer Assessment of Motor Performance and Sensory Assessment (Polish version, FMA-PL) from the original English version into Polish. In the next step, the draft was back-translated by two independent translators in accordance with the guidelines proposed by the WHO and Guillemin [31,32]. The whole adaptation procedure is presented in Figure 1. The next stage was the establishment of a panel of judges. These were specialists experienced in their field, fluent in English, taking internships and courses abroad, as well as working with patients with neurological deficits at various stages of improvement: physicians, physiotherapists, and a neuropsychologist. During the judges’ panel, the jurors analyzed several differences resulting from the ambiguous interpretation of some English words and phrases that appeared during the translations. The judges were tasked with reviewing all versions of the translations, the original version of the tool, and, after making corrections, determining the final version, paying attention to the graphic design, layout, order of questions, and instructions, which should not differ from the original version. A further stage in the procedure was to conduct studies on a group of stroke patients using an outcome measure.

### 2.4. Procedure

The FMA-PL was carried out by two experienced neurological physiotherapists who were trained in the administration of this outcome measure. Reproducibility, that is, the degree to which the score is free from random error, was assessed with test–retest and inter-rater procedures. To obtain measures of inter-rater reliability, two raters independently examined the patients at the same time in a quiet hospital room. Test–retest reliability was obtained by the same observer, who examined the patients twice a day with a two-hour gap between assessments [33,34]. The results were collected for the total and subscales of the FMA-PL [35,36]. The assessment of motor and sensory activity was carried out in accordance with the instructions described by Sullivan et al. [29].

### 2.5. Statistical Analysis

All measurements were statistically analyzed using TIBCO Software Inc. (2017) Statistica, version 13 (TIBCO Software Inc., Palo Alto, CA, USA), which aimed to determine the internal consistency of the test in individual researchers, as well as to determine the degree of compatibility of measurements between judges, in order to determine whether the Polish-language outcome measure is a good tool for assessing stroke patients.

### 2.6. Reliability

For the assessment of the test–retest and inter-rater consistency of measurement results, the interclass correlation coefficient (ICC) was selected, which can be used when measures are performed by several researchers. In accordance with Partney and Watkins (1993), the ICC results were interpreted as follows: 1.0–0.76 excellent–high repeatability; 0.75–0.51 good repeatability; 0.50–0.26 average–low repeatability; 0.25–0.00 no repeatability [37].

The consistency of the results was also assessed on the basis of an analysis of the standard error of measurement (SEM) value and the minimum detectable change (MDC). The SEM determines to what extent the values of the measure will differ on subsequent measurements under the same conditions. However, the minimum detectable change determines the smallest difference between the two measurements, which results (with a 95% confidence level) from the actual rather than random fluctuations of the measurement [38,39]. According to the following formulas, where $r_{xx}$ is the reliability of the test, $S_{x}$ is the standard deviation of all the scores.

$$SEM=S_{x}\sqrt{1-r_{xx}}$$

MDC=1.96×SEM×√2

### 2.7. Internal Consistency

The internal consistency of the test was determined by Cronbach’s alpha indicator. The value of the indicator is between 0 and 1. This indicator can also show which of the questions reduces the reliability of the test. The higher the value, the greater the reliability of the scale. It is assumed that values above 0.7 indicate the correct reliability of the scale [40].

## 3. Results

### 3.1. Patients’ Clinical Characteristics

The study involved 86 stroke patients, including 56 males (65%) and 30 females (35% of all persons being tested). The mean age of patients was 64 ± 12 years. None of the subjects were extremely obese or emaciated. All patients underwent ischemic stroke and were undergoing neurological rehabilitation during the study. The full characteristics of the subjects are shown in Table 1.

Three domains were evaluated on the FMA-PL scale. From the entire test, results were obtained in the range of 12–124 points: 64 points for FMA-UE-PL 2, 34 points for FMA-LE-PL 4, and 24 points for FMA-S-PL 0. All results divided into individual questions in their domains are included in Table 2.

### 3.2. Reliability

ICC values were calculated, with one rater on two trials (test–retest, Table 3) and two raters at the same time (inter-rater, Table 4). Excellent reliability was achieved for the entire questionnaire and for individual domains. The SEM and MDC of the total FMA-PL score calculated for test–retest measurements were *0.22 and 1.60 points,* respectively. For inter-rater measurements, the SEM and MDC for the total FMA-PL score amounted to *1.27 and 3.53 points,* respectively.

### 3.3. Internal Consistency

The results for the internal consistency of the test were presented using Cronbach’s alpha indicator for three measurements taken by two raters. Table 2 shows the point scores and Cronbach’s alpha indicator values for all domains and tasks in each domain. For the total FMA-PL result, the results did not differ between measurements, and internal consistency was very high, with a value of *0.938–0.939*. Similarly, a high consistency rate of *0.932–0.934* was achieved for the f domain. In this domain, for task 7, the rate was the lowest at *0.912–0.914*. The lower consistency was for the FMA-LE-PL domain, which ranged between *0.790* and *0.795*. In this domain, for task 6, the indicator value was the lowest, at *0.682–0.693*. The FMA-S-PL domain had the lowest consistency rate compared to the results of the entire FMA-PL and the FMA-UE-PL and FMA-LE-PL domains. Cronbach’s alpha value was *0.634–0.722*. For individual tasks in the domain, the rate was higher.

## 4. Discussion

This study showed the process of the translation of the English version of the original FMA into the Polish language, then its adaptation and determination of reliability. The results of this work will be important in the clinical practice of physiotherapists, physicians, and occupational therapists. The newly adapted outcome measure with a high clinical recommendation will be a valuable tool for assessing Polish stroke patients. The FMA-PL is an accurate, responsive, easy-to-perform outcome measure that can be used without any special expensive equipment in a variety of clinical settings. This outcome measure will allow, in a very objective and uniform way, to determine the functional capacity of the patient and set short- and long-term goals in the process of improving the physical condition of Polish patients with stroke. It can be an opportunity to perform an international exchange of the patient’s clinical evaluation results after a stroke. The high clinical recommendation of the FMA-UE and FMA-LE is evident. The FMA, compared to other tools such as the Stroke Rehabilitation Assessment of Movement (STREAM) [41], the Action Research Arm Test (ARAT) [42], and the Wolf Motor Function Test [43], has the highest clinical recommendation. It evaluates the motor function of both the upper and lower limbs comprehensively. The sensory assessment domain does not have such a good clinical recommendation, but it has educational impact. The FMA-PL is the only clinical utility scale for stroke patients with such a high recommendation translated into Polish. Currently, the original Fugl-Meyer scale is officially translated and published in Spanish (FMA-LE 60–100%, FMA-UE 50–100% inter-rater agreement), Danish (FMA-UE ICC = 0.95), Italian (FMA-UE 70–100% inter-rater agreement), Romanian (FMA-UE ICC = 0.98), and Brazilian Portuguese (FMA-UE ICC = 0.98, FMA-LE ICC = 0.90, for movement sense ICC = 0.98, and upper and lower-limb passive range of motion ICC = 0.84 and 0.90, respectively). However, this is the first translation of the scale in three domains (except Brazilian Portuguese where all domains have been translated). It is also worth highlighting the fact that a large number of respondents were assessed. Other studies were conducted on 10 to 31 stroke patients [15,17,18,19,44,45]. The largest group of respondents was collected in a study conducted by Hernández et al. [16], where the outcome measure was translated into Spanish. In the above study, no pilot study was conducted on a small group of patients, as in the studies of Barbosa et al. [15] and Michaelsen et al. [45]. However, a well-described procedure of translation and adaptation of the FMA-PL with its results was presented. In the discussion panel, not only the judges with clinical experience attended but also researchers and physiotherapists with neurological specializations. 

A high level of internal consistency was observed in a large group of respondents in the FMA-UE-PL and FMA-LE-PL domains and was also good in the FMA-S-PL domain. In other studies, high internal consistency and compliance were observed in much smaller trials, which is why they cannot be compared [15,17,18,19,44,45]. A study assessing compliance in a comparable population was carried out only for the FMA-UE domain and also achieved high compliance [16]. A higher internal consistency of the FMA-S domain than in this study was presented for a population of 38 by Platz et al. [46]. A study assessing the internal consistency as well as compliance of researchers for the sensorimotor evaluation of FMA in limbs was presented for the first time on such a large population. Earlier, Michaelsen et al. [45] presented the results of the translation and adaptation of almost all domains in the scale but on a population of 18 patients.

## 5. Limitations

The main limitation of the study was differences in the severity of stroke and the rehabilitation programs of the patients in the study group. However, at the time of the study, we had limited access to a more homogenous group of stroke survivors. The study used a 2 h test–retest interval. Although this approach is justified because such solutions have been used before in similar studies, this interval is relatively short and may have significant consequences in the form of patient fatigue and memory effects [47,48,49]. Future research with the FMA-PL should determine its validation. Comparison with other gold-standard outcome measures would assess if the tool is valid and may be used among Polish stroke survivors.

## 6. Summary

It can be concluded that the FMA-PL is a coherent and reliable outcome measure for sensory and motor evaluation of the upper and lower limbs in subacute and chronic stroke patients among the Polish population. This study confirms the reasonable use of the outcome measure in observations and clinical and scientific trials. It is worth highlighting the educational value of the FMA-PL for the medical profession. This allows us to obtain a Polish-speaking, universal, specific outcome measure for assessing the motor and sensory condition of a stroke patient.

## Figures and Tables

**Table 1:** Demographics and characteristics (*n* = 86).

Characteristics	
Gender (*n*) (male/female)	86 (56/30)
Age (years), mean (SD)	64 (12)
BMI (kg/m^2^), mean (SD)	28 (5)
Side of stroke (*n*) (right/left)	36/50
Time since stroke (weeks), mean (SD)	6 (6)
Accompanying diseases (*n*):	
Hypertension (%)	44 (51.2)
Diabetes	22 (25.6)
Shoulder pain	10 (11.6)
Thyroid disease	8 (9.3)
Myocardial infarction	4 (4.7)
other heart conditions	8 (9.3)
Hypercholesterolemia	3 (3.5)
Respiratory system diseases	3 (3.5)
FMA-UE-PL (0–66), mean (SD)	51.6 (21.2)
FMA-LE-PL(0–34), mean (SD)	26.4 (8.9)
FMA-S-PL (0–24), mean (SD)	21.2 (6.1)
Total FMA-PL(0–124), mean (SD)	99.4 (33.3)

FMA—Fugl-Meyer Assessment; UE—upper extremity; LE—lower extremity; S—sensory function; FMA-PL—Fugl-Meyer Assessment of Motor Performance and Sensory Assessment (Polish version); mean (SD)—standard deviation.

**Table 2:** Results of Cronbach’s alpha.

Variable	Result			Result of Cronbach’s Alpha		
	Expert 1	Expert 2	Expert 2 in 2 Time	Expert 1	Expert 2	Expert 2 in 2 Time
Total FMA-PL	99.2 ± 33.3	99.5 ± 33.6	99.5 ± 33.6	0.939	0.940	0.940
FMA-UE-PL	51.6 ± 21.2	51.5 ± 21.5	51.5 ± 21.5	0.934	0.933	0.933
FMA-UE-PL 1 Reflex activity	3.84	3.80	3.80	0.949	0.946	0.946
FMA-UE-PL 2 Flexor synergy	9.67	9.69	9.69	0.915	0.914	0.913
FMA-UE-PL 3 Extensor synergy	5.05	5.09	5.09	0.923	0.922	0.922
FMA-UE-PL 4 Movement combining synergies	4.91	4.92	4.92	0.921	0.920	0.919
FMA-UE-PL 5 Movement out of synergies	4.66	4.66	4.66	0.919	0.920	0.919
FMA-UE-PL 6 Normal reflex activity	1.13	1.17	1.17	0.939	0.938	0.937
FMA-UE-PL 7 Wrist	7.34	7.30	7.30	0.915	0.914	0.913
FMA-UE-PL 8 Hand	10.76	10.66	10.65	0.927	0.928	0.928
FMA-UE-PL 9 Coordination and speed	4.21	4.22	4.21	0.923	0.921	0.921
FMA-LE-PL	26.4 ± 8.9	26.7 ± 8.9	26.7 ± 9.0	0.790	0.794	0.795
FMA-LE-PL 1 Reflex activity	3.8	3.8	3.8	0.823	0.823	0.824
FMA-LE-PL 2 Flexor synergy	11.3	11.3	11.3	0.745	0.750	0.754
FMA-LE-PL 3 Extensor synergy	3.4	3.4	3.4	0.729	0.730	0.732
FMA-LE-PL 4 Movement combining synergies	2.7	2.8	2.8	0.723	0.718	0.719
FMA-LE-PL 5 Normal reflex activity	0.8	0.9	0.9	0.800	0.815	0.814
FMA-LE-PL 6 Coordination/speed	4.5	4.5	4.5	0.683	0.690	0.693
FMA—S-PL	21.2 ± 6.1	21.3 ± 6.2	21.3 ± 6.2	0.634	0.723	0.722
FMA—S-PL 1 Light touch	7.2	7.1	7.1	0.937	0.938	0.938
FMA—S-PL 2 Position	14.1	14.2	14.2	0.939	0.939	0.939

FMA—Fugl-Meyer Assessment; UE—upper extremity; LE—lower extremity; S—sensory function; FMA-PL—Fugl-Meyer Assessment of Motor Performance and Sensory Assessment (Polish version).

**Table 3:** Test–retest reliability, *n* = 86.

		ICC	SEM	MDC
FMA-UE expert 2	FMA-UE expert 2 in 2 time	0.999	0.19	0.52
FMA-LE expert 2	FMA-LE expert 2 in 2 time	0.999	0.15	0.42
FMA-sensation expert 2	FMA-sensation expert 2 in 2 time	1.000	0.00	0.00
Total FMA expert 2	Total FMA expert 2 in 2 time	0.999	0.21	0.60

FMA—Fugl-Meyer Assessment; UE—upper extremity; LE—lower extremity; ICC—intraclass correlation coefficient; SEM—standard measurement error; MDC—minimal detectable change.

**Table 4:** Inter-rater reliability, *n* = 86.

Variable	Lower CI (95%)	ICC	Upper CI (95%)	SEM	MDC
FMA-UE-PL	0.996	0.997	0.998	1.11	3.07
FMA-LE-PL	0.988	0.992	0.994	0.81	2.26
FMA—S-PL	0.997	0.998	0.998	0.29	0.82
Total FMA-PL	0.998	0.999	0.999	1.27	3.53

FMA—Fugl-Meyer Assessment; UE—upper extremity; LE—lower extremity; S—sensory function; FMA-PL—Fugl-Meyer Assessment of Motor Performance and Sensory Assessment (Polish version); CI—confidence interval; ICC—intraclass correlation coefficient; SEM—standard measurement error; MDC—minimal detectable change.

## Data Availability

The data presented in this study are available on request from the corresponding author.
